# 3-Aminopropyl-triethoxysilane-Functionalized Tannin-Rich Grape Biomass for the Adsorption of Methyl Orange Dye: Synthesis, Characterization, and the Adsorption Mechanism

**DOI:** 10.1021/acsomega.2c02101

**Published:** 2022-05-23

**Authors:** Edmo H. M. Cavalcante, Iuri C. M. Candido, Helinando P. de Oliveira, Kamilla Barreto Silveira, Thiago Víctor de Souza Álvares, Eder C. Lima, Mikael Thyrel, Sylvia H. Larsson, Glaydson Simões dos Reis

**Affiliations:** † Institute of Materials Science, Federal University of Sao Francisco Valley, Juazeiro 48920-310, BA, Brazil; ‡ Institute of Chemistry, Federal University of Rio Grande do Sul (UFRGS), Av. Bento Gonçalves, Porto Alegre 9500, Rio Grande do Sul, Brazil; § Swedish University of Agricultural Sciences, Department of Forest Biomaterials and Technology, Umeå 90183, Sweden

## Abstract

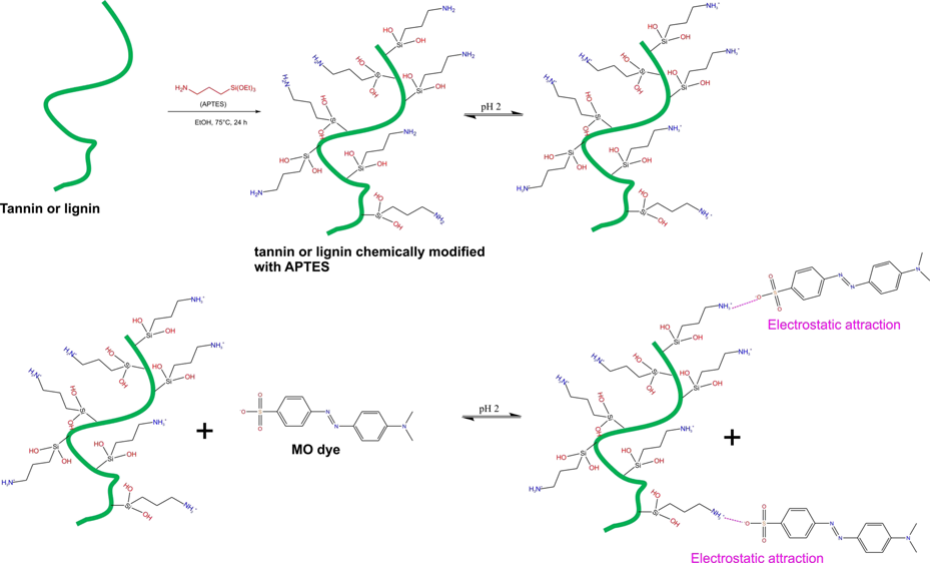

A biomass amino silica-functionalized material was successfully prepared by a simple sol–gel method. 3-Aminopropyltriethoxysilane (APTES) was added to a tannin-rich grape residue to improve its physicochemical properties and enhance the adsorption performance. The APTES functionalization led to significant changes in the material’s characteristics. The functionalized material was efficiently applied in the removal of methyl orange (MO) due to its unique characteristics, such as an abundance of functional groups on its surface. The adsorption process suggests that the electrostatic interactions were the main acting mechanism of the MO dye removal, although other interactions can also take place. The functionalized biomass achieved a very high MO dye maximum adsorption capacity (*Q*_max_) of 361.8 mg g^–1^. The temperature positively affected the MO removal, and the thermodynamic studies indicated that the adsorption of MO onto APTES-functionalized biomass was spontaneous and endothermic, and enthalpy is driven in the physisorption mode. The regeneration performance revealed that the APTES-functionalized biomass material could be easily recycled and reused by maintaining very good performance even after five cycles. The adsorbent material was also employed to treat two simulated dye house effluents, which showed 48% removal. At last, the APTES biomass-based material may find significant applications as a multifunctional adsorbent and can be used further to separate pollutants from wastewater.

## 1 Introduction

With the fast industrialization, there is a need to produce colored products, and the volume of wastewater containing colors has increased globally.^1,2^ Pigments and dyes are organic compounds with a wide variety of colors, either natural or synthetic, employed in many activities in the textile, cosmetic, medical, and food industries.^1,2^ Due to the extensive use of pigments and dyes, these compounds are commonly found in these industries’ wastewaters and often found in natural water.^1−3^

The global pigment and dye market was evaluated at USD 33.2 billion in 2019.^4^ The azo dyes (containing the −N=N– group) are the leading group of synthetic dyes which are major environmental contaminants.^1−3^ For example, methyl orange (MO) is an anionic dye, and it belongs to a class of azo dyes, presenting aromatic rings and azo groups. Due to its chemical characteristics, MO is highly toxic, teratogenic, carcinogenic, and harmful to living organisms.^5,6^ Moreover, these dyes can decrease the dissolved oxygen in the stream and destroy aquatic life via biological and chemical changes.^1−3^ Thus, it is imperative that they must be properly treated before discharging effluents in natural water. MO is an anionic dye that was chosen as an adsorbate in this study.

Different techniques are employed to remove MO dye from solutions, including ultrafiltration,^7^ advanced oxidation processes,^8^ electrochemical degradation,^9^ photocatalytic degradation,^10^ and coagulation.^11^ Although these techniques reach good efficiency of removal, they present serious drawbacks such as high implementation and operational costs and management complexity.^11^ However, the adsorption treatment method is adequate for dye removal from wastewater because of its low cost, easy operating conditions, and high efficiency of the adsorption procedure.^12,13^

Selecting the adsorbent is a crucial step in designing an effective adsorption process. Many adsorbents are available in the literature for efficient dye removal, including activated carbons,^14−16^ chitosan composites,^12,13^ metal–organic framework (MOF) materials,^17^ porous silica materials,^18−22^ carbon nanotubes,^23^ graphene oxide,^24^ and so forth. However, these materials have several disadvantages, including complex synthesis routes and extremely high costs, limiting their application in adsorption processes.

To surpass these issues, adsorbents based on plant biomass have been successfully utilized as cost-effective adsorbents for water treatment.^25^ Furthermore, applying biomass residues without pyrolysis makes the adsorption process very affordable and more environment-friendly.^25^ Biomass is one of the most abundant renewable organic raw materials on earth. However, literature shows that biomass materials tend to exhibit a high adsorption capacity for pollutant species after suitable chemical modification.^25^ Based on this, many suitable routes have been reported to modify the biomass surface/structure by grafting with chemical moieties, including hexadecyltrimethylammonium bromide,^26^ organic polymers,^27^ and 3-aminopropyltriethoxysilane (APTES).^28−30^

APTES is an organic silane compound that efficiently couples with oxygen atoms and hydroxyl groups present on silicate^31,32^ and biomass precursors.^28−30^ Recently, many works have reported the formation of hybrids/composite materials with APTES biomass-based materials.^28−30^ In this context, it is vital to produce new APTES biomass for efficient application in removing anionic and other types of dyes from aqueous effluents.

This work used grape winery waste (GWW) as a feedstock for APTES functionalization. Grape wine represents one of the most important alcoholic beverages globally with a continuously growing demand.^33^ The annual production worldwide can reach almost 70 million tons, and around 80% of the processed grapes are used for wine production, whereas 20% of the processed grapes remain as pomace.^33^ Only in the Mediterranean countries, the annual production of grape pomace can be as high as 1200 tonnes per year.^33^ Therefore, utilizing this biomass residue in the production chain is important to decrease the pollution caused by dyes and add value to the chemically modified biomass besides reducing the availability of dyes in the environment.

The present study provides a facile synthesis pathway to obtain an efficient GWW biomass-based adsorbent for MO dye removal. GWW was functionalized using APTES, forming a GWW-APTES-grafted material.^28−30^ Most studies focused on APTES functionalization are related to inorganic materials such as silica and alumina silicate materials.^18,19,22^ Research related to the effect of APTES functionalization on biomass precursors is lacking. Therefore, the effect of APTES functionalization on physicochemical properties of GWW-APTES was fully investigated. The grafting of APTES is performed to boost the adsorption performance of the modified material. Nevertheless, the role of the modification process on the MO adsorption of GWW-APTES is fully elucidated, where important interactions can occur between MO and −NH_2_ groups present on the GWW-APTES surface. Comprehensive physicochemical characterizations including scanning electron microscopy (SEM)-energy-dispersive spectroscopy (EDS), Fourier transform infrared (FTIR) spectroscopy, X-ray diffraction (XRD), X-ray photoelectron spectroscopy (XPS), and point of zero charge were performed to provide valuable insights into the material’s properties. Moreover, the behavior of GWW-APTES toward MO was examined in terms of the pH effect, kinetic studies, equilibrium isotherms, and effluent treatment.

## 2 Experimental Section

### 2.1 Biomass Precursor and Reactants

The grape biomass residues were obtained from COANA (Petrolina, Brazil). The biomass was milled at a maximum particle size of 200 mesh. APTES (98%) and MO were purchased from Merck (Brazil) and were utilized as received. Deionized water was used during the entire investigation.

### 2.2 Synthesis of GWW/APTES

**Figure 1:**
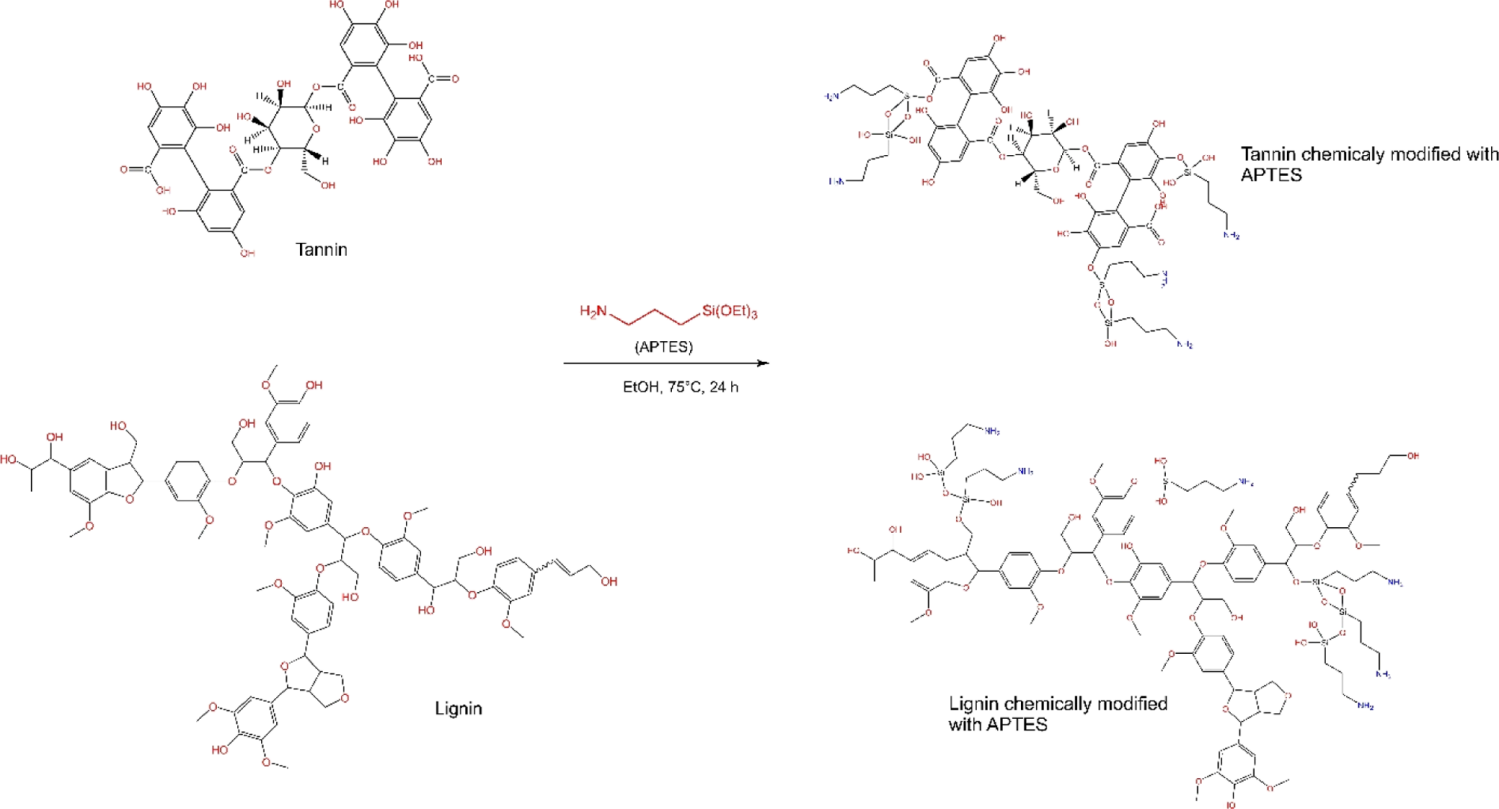
Grafting of APTES with GWW (lignin and tannin parts).

Modified GWW (GWW/APTES) was produced by the grafting method as previously described.^28−30^ First, 3 g of GWW was added to 50 mL of ethanol together with 3 g of APTES (ratio 1:1, w/w). Next, the mixture was magnetically stirred under reflux (80 °C) for 24 h until the formation of a brown product. This procedure allows the successful synthesis of a grafted material in a single step. After the reaction, the sample was washed multiple times with ethanol and water to remove the nongrafted APTES on the GWW-APTES surface. The synthesis route of the GWW-APTES adsorbent is shown in Figure 1.

### 2.3 Analyses

Spectroscopy measurements of the MO dye solutions were performed using a Hach UV–vis spectrometer model DR500. The SEM images were acquired using a scanning electron microscope (Vega3XM Tescan) using an acceleration voltage of 20 kV. The KBr method was used to acquire the FTIR spectra (IR Prestige-21Fourier Shimadzu, Kyoto, Japan). XPS spectra were collected using a Kratos Axis Ultra DLD electron spectrometer using a monochromated Al Kα source operated at 150 W. An analyzer pass energy of 160 eV for acquiring survey spectra and a pass energy of 20 eV for individual photoelectron lines were used. The binding energy scale was calibrated following the ASTM E2108 and ISO 15472 standards. Processing of the spectra was performed with the Kratos software. The zeta potential was measured using a Nano ZS apparatus (Malvern PCS Instruments, UK) using the Smoluchowski model with samples disposed at 25 °C in ultra-purified water with experiments performed in triplicate.

### 2.4 Adsorption Studies

The adsorption tests were carried out using MO dye as an adsorbate ranging from 50 to 2000 mg L^–1^. In addition, the effect of pH (from 2.0 to 10.0) was also evaluated on MO removal. MO solution aliquots of 20 mL were placed in Falcon tubes (50.0 mL) containing a mass of the adsorbent varying from 20 to 100 mg. The kinetic tests were performed by shaking the tubes with GWW-APTES and solutions at 150 rpm, varying the contact time from 1 to 400 min. After shaking, the solid adsorbent and the liquid phase were separated by centrifugation. Then, with a pipette, the working solution amounts were withdrawn to quantify the remaining MO dye concentration in the solution using UV–vis spectroscopy at λ_max_ of 465 nm. The adsorption capacity (eq 1) and the percentage of MO dye removal (eq 2) are calculated as given below
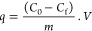
1
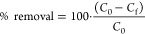
2where *m* is the mass of GWW/APTES (g); *C*_0_ and *C*_f_ are the initial and final MO dye concentrations (mg L^–1^), respectively; *q* is the adsorption capacity of the MO dye uptaken by GWW/APTES (mg g^–1^); and *V* is the volume of the MO dye solution (L).

### 2.5 Studies of Adsorption Kinetics, Equilibrium, and Thermodynamics

The pseudo-first-order (PFO; eq 3), pseudo-second-order (PSO; eq 4), and general-order (GO; eq 5) kinetic models^34^ were fitted to the kinetic data.
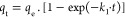
3
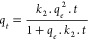
4
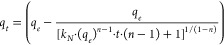
5

The Langmuir (eq 6), Freundlich (eq 7), and Liu (eq 8) isotherm models were utilized to fit the equilibrium data.^34^
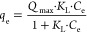
6
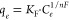
7
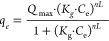
8

The Gibb’s free energy change (Δ*G*^0^, kJ mol^–1^), enthalpy change (Δ*H*^0^, kJ mol^–1^), and entropy change (Δ*S*^0^, J mol^–1^ K^–1^) were evaluated using eqs 9–12, respectively.^35−37^
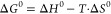
9
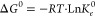
10
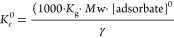
11

The combination of eqs 9 and 10 leads to eq 12.
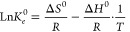
12

The kinetic, equilibrium, and thermodynamic equations are further explained in the Supporting Information.^34−37^

The quality control of adsorption data is further described in the Supporting Information.

### 2.6 Synthetic Effluents

Treatment of colorful synthetic effluents was performed using an approach suggested elsewhere^23,26^ and is further explained in the Supporting Information.

### 2.7 GWW/APTES Regeneration Tests

For regeneration tests, MO dye-laden GWW/APTES was washed with water to remove any unadsorbed dye and dried overnight in an oven at 50 °C. The dried-laden GWW/APTES was contacted with a 1 M NaOH eluent and agitated for 4 h. The desorbed MO dye was then separated from GWW/APTES.

## 3 Results and Discussion

### 3.1 Materials Characterization

#### 3.1.1 Morphology of Materials

**Figure 2:**
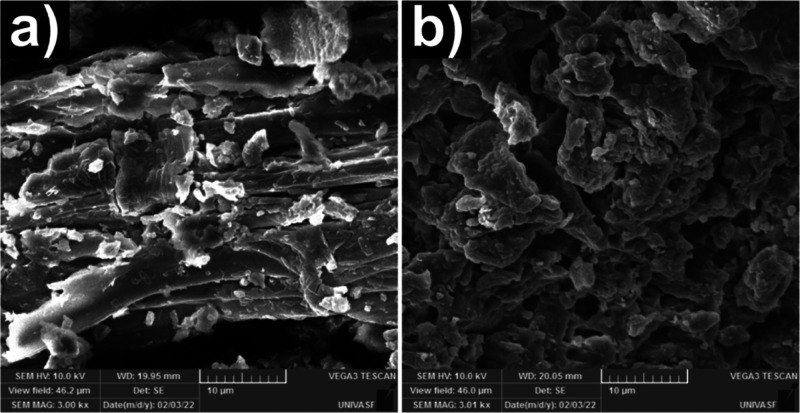
SEM images of GWW (a) and GWW/APTES (b).

Figure 2 shows the morphology of the nonmodified (a) and modified (b) biomass with APTES. Remarkable differences are observed in the materials’ morphology. The nonmodified biomass displays a more fibrous-like structure with some roughness on its surface. After APTES modification, the fibrous surface disappeared, and its surface became extremely rough with some agglomerated material with the modified sample also showing some superficial holes and cavities.

The remarkable difference between both samples could be related to the bonding between silica and biomass structures that takes place during the sol–gel process that encapsulates the biomass material.^24,28^ Thus, based on SEM images, it is possible to infer that APTES successfully modified the grape biomass surface, which might have huge benefits in adsorbing pollutants in water.

To corroborate the SEM analysis, the EDS mapping of the modified sample is shown in Supporting Information, Figure 1. The image shows that the material’s surface is covered by silicon, and nitrogen is also identified in the elemental mappings; these images corroborate the results of the SEM analysis.

#### 3.1.2 FTIR, XRD, and XPS Analyses

**Figure 3:**
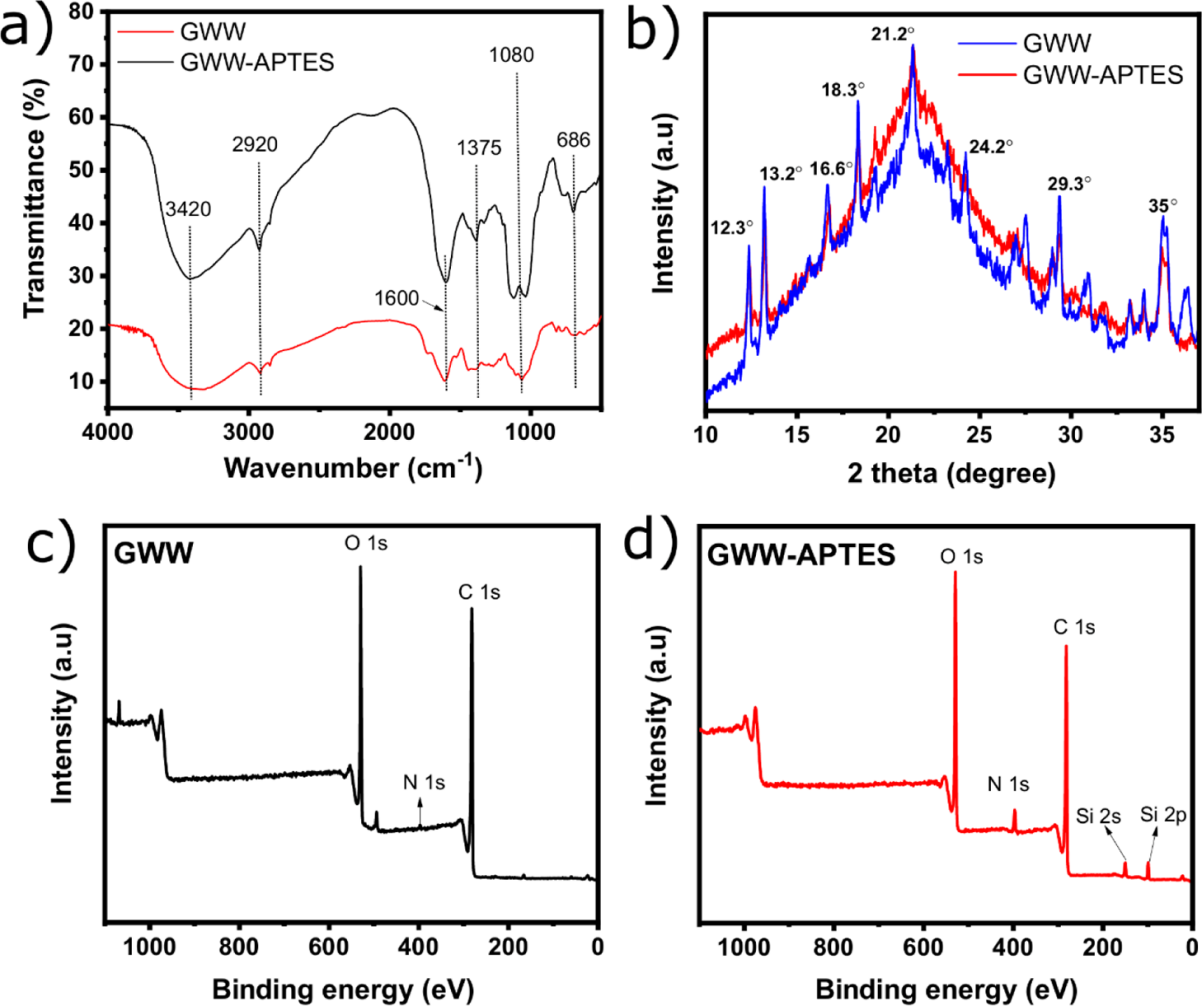
FTIR spectra of GWW and GWW/APTES (a), XRD patterns of GWW and GWW/APTES (b), and XPS survey scans of GWW (c) and GWW/APTES (d).

FTIR analysis was carried out to observe the effect of APTES functionalization on the biomass surface functional groups. It provides useful information about the chemical surface activity of the adsorbents that, in turn, might reflect better adsorption properties. The FTIR spectra of GWW and GWW/APTES are presented in Figure 3a. Band assignments of the spectrum of GWW/APTES indicate that the functionalization process successfully grafted new functional groups on GWW’s pristine form. Besides, it is observed that the spectrum of GWW/APTES has much wider bands and more prominent peaks.

For instance, the new band at 686 cm^–1^ can be attributed to the region of angular deformation outside the plane of N–H (δ NH_2_), referred to as the amino groups present in APTES.^28^ Another new peak at 1375 cm^–1^ was assigned to the C–N group that also comes from APTES.^28,31,32^

The other peaks and bands observed in both materials are related to the functional groups commonly presented in biomass-derived materials.^28,30^ For instance, the broad band at 3420 cm^–1^ is assigned to O–H stretching vibrations^28^ and that at 2920 cm^–1^ can be attributed to C–H stretching vibrations; note that the peak is slightly sharper in GWW/APTES, which could be the contribution of APTES modification because it has many CH groups in its structure (see Figure 1). The two peaks at 1600 and 1080 cm^–1^ can be related to carboxyl groups (HO–C=O) and C–O stretching vibration, respectively.^21,28^ It can also be observed that both peaks are bigger in GWW/APTES than in GWW, which could be a reflex of functionalization.

The new bands and their higher intensities in GWW/APTES successfully confirm the surface modification of the grape biomass into a material with an abundance of functional groups on its surface, which in turn can boost its adsorption properties once functional groups exert a vital role in the overall adsorption process.

XRD was used to study the amorphous and/or crystalline phases of GWW and GWW/APTES. The XRD patterns of nonmodified and modified samples are shown in Figure 3b. The XRD patterns of nonmodified and modified samples show important differences. GWW shows several more peaks and wider peaks than GWW/APTES, highlighting its semicrystalline or paracrystalline phases,^30^ confirming the crystalline character of the substrate material. For instance, the main peaks could be related to the crystalline cellulose, much more prominent in GWW.^38^ On the other hand, GWW/APTES suggested the formation of an amorphous material and, consequently, successfully modified GWW into GWW/APTES. The literature shows that APTES has a more amorphous character.^29,30^ However, it is also observed that some crystalline peaks in GWW/APTES highlight both characteristics of amorphous and crystalline phases.

The surface chemical composition of GWW and GWW/APTES was evaluated by XPS analysis (Figure 3c,d). The survey scan of GWW/APTES showed signals at 150 and 100 eV, which correspond to the binding energies of Si 2s and Si 2p orbitals, respectively; no Si signals were observed in GWW, which strongly indicates that GWW/APTES was successfully modified by APTES. The signals for the C 1s and O 1s orbitals of carbon and oxygen are observed at 284 and 530 eV, respectively. The higher N 1s orbital signal at 400 eV of GWW/APTES is evident. The quantitative information from XPS is shown in Table S2. The carbon content is higher in GWW (76.1%) compared to that in GWW/APTES (69.5%), while oxygen and nitrogen are higher in GWW/APTES. As shown in the survey spectra, silicon was detected only in GWW/APTES (4.3%). These results confirm the successful functionalization of GWW by APTES.

#### 3.1.3 Zeta Potentials and Isoelectric Points

The pH_pzc_ of GWW/APTES was determined and discussed as a point where the material’s surface has potential charges equal to zero. It means, for the pH values higher than pH_PZC_, the adsorbent presents a negative surface charge while lower values the surface of the adsorbent presents positive surface charge..^27^ This characteristic has been used so far to understand the surface chemistry of materials and the mechanism of their interaction with other molecules in polar solvent media.

**Figure 4:**
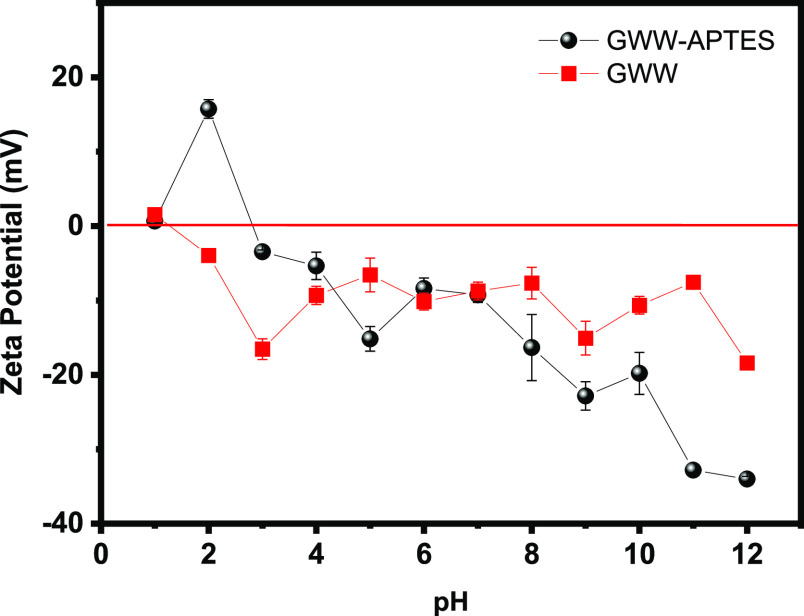
Zeta potentials of GWW and GWW/APTES at different pH values.

The change in the zeta potential values of GWW/APTES as a function of suspension medium pH is presented in Figure 4. As observed, the overall zeta potentials gradually decreased to more negative values with increasing pH. Both GWW and GWW/APTES possess a negative zeta potential across most of the pH range.^28^ This behavior continues until the zeta potential reaches the isoelectric point at a pH of 1.2 and 2.8 for GWW and GWW/APTES, respectively; this finding means that the grafting of APTES successfully modified the GWW surface. Such a smooth shift to a less negative potential with decreasing pH indicates a more appreciable anionic character of the GWW/APTES surface. De Souza et al.^39^ prepared a composite from green iron oxide nanoparticles and *Camellia sinensis* (black tea) and further functionalized with APTES. It was found that the isoelectric point of the functionalized composite was at pH 1.9, which was later successfully employed for anionic azo dye removal. Therefore, our results are backed up by the literature values.

### 3.2 Adsorption Results

It is highlighted that preliminary adsorption tests were performed using GWW in the pristine form, and some issues were detected, including very low stability under both basic and acid pH, and even at neutral pH, GWW released color into water. This can affect the accuracy of the UV–vis results.

In addition, the adsorption capacity of the GWW material was extremely low compared to that of GWW/APTES. Due to the above reasons, we believe that they do not justify its use in further adsorption tests (pH effect and kinetic and equilibrium studies). Therefore, based on these reasons, only the GWW/APTES adsorbent was utilized in the subsequent adsorption experiments of the MO dye.

#### 3.2.1 pH Studies

**Figure 5:**
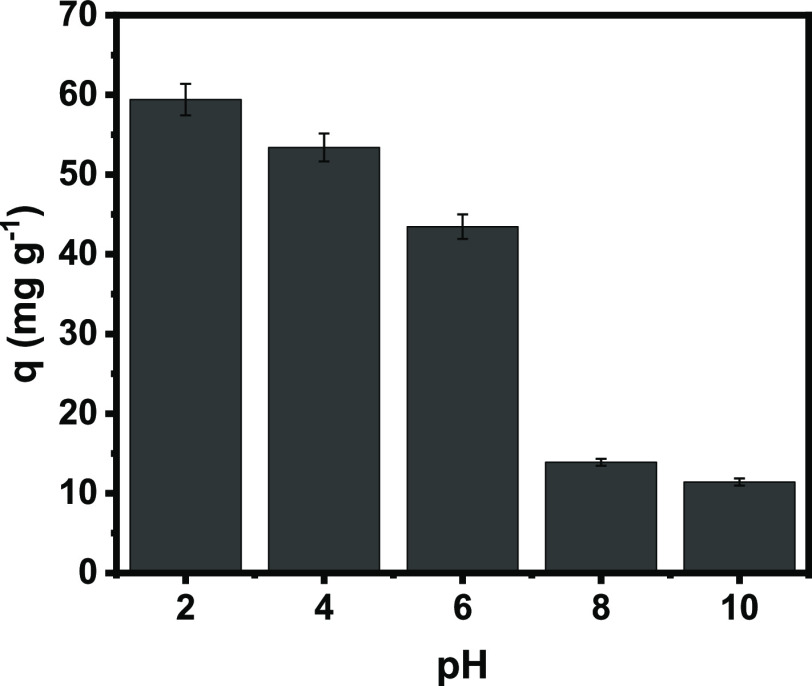
Effect of initial pH of the MO dye on adsorption onto GWW/APTES. Conditions: 25 °C, an adsorbent dosage of 2.0 g L^–1^, and a contact time of 6 h.

The pH of the adsorbate solution plays a vital role in controlling the adsorption process; the pH of the solution influences the surface charge of the adsorbent. The effect of pH on MO removal at different pH values is shown in Figure 5. Batch experiments were carried out in the pH range of 2–10, using an initial MO dye concentration of 200 mg L^–1^, to study the effect of pH on the MO removal performance of GWW/APTES.

Figure 5 shows that the highest MO adsorption capacities of GWW/APTES occurred in solutions with acidic pH (with the highest value at pH 4 with a *q* of 62.4 mg g^–1^). Our results are in line with the literature that frequently reports that the optimum pH values for MO removal are between 2 and 6.^28,40−43^ At a low pH, the strong electrostatic attraction occurs between the positively charged surface of GWW/APTES and the negatively charged MO dye due to the ionization of functional groups (amines) of GWW/APTES and MO molecules. This suggests that the main adsorption mechanism for MO onto GWW/APTES was electrostatic attraction because the pH value highly influences it.

Even the pH is an important variable that boosts the MO dye adsorption; under acidic pH, changing the pH of the solution or effluent increases the operational cost of the process to a large extent, which is not always justified; therefore, the pH of the MO solutions was maintained (at around 6.0–6.2) for the further experiments.

#### 3.2.2 Kinetic Studies

**Figure 6:**
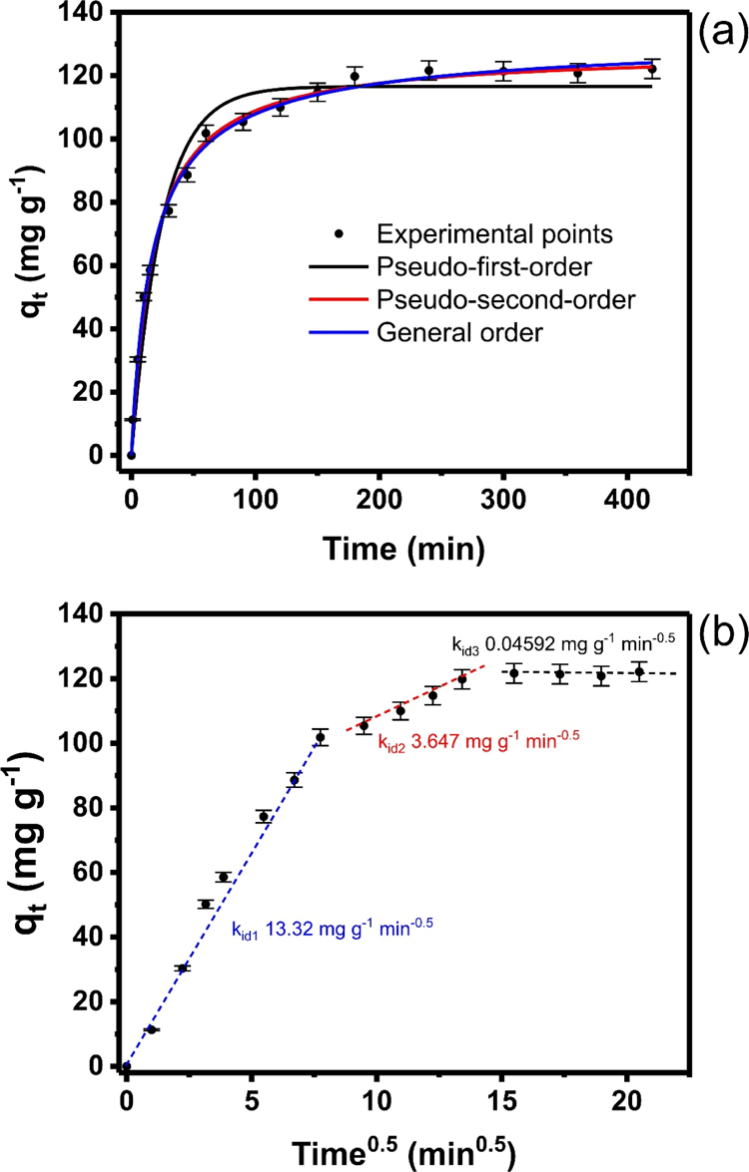
Kinetic (a) and intraparticle diffusion (b) curves of MO adsorption onto GWW/APTES. MO dye had an initial concentration of 200 mg g^–1^, temperature 25 °C, the mass of adsorbent 2.0 g L^–1^, and a pH of 2.0.

Kinetics is an important study for elucidating the mechanism that takes place during the adsorption process, such as mass transport processes and diffusion control. Therefore, three models, PFO, PSO, and GO, were applied to evaluate the kinetics of MO adsorption on GWW/APTES. The contact time up to 400 min at an initial concentration of 400 mg L^–1^ of MO was evaluated in the kinetic process, and its curve and parameters are shown in Figure 6 and Table 1, respectively.

**Table 1:** Kinetic Fitting Parameters

kinetic models	
Pseudo-first-order	
*q*_e_ (mg g^–1^)	116.6
*k*_1_ (min^–1^)	0.04075
*t*_1/2_ (min)	17.01
*t*_0.95_ (min)	73.52
*R*^2^ adjusted	0.9749
SD (mg g^–1^)	6.642
BIC	66.77
Pseudo-second order	
*q*_e_ (mg g^–1^)	127.8
*k*_2_ (g mg^–1^ min^–1^)	4.509 × 10^–4^
*t*_1/2_ (min)	16.03
*t*_0.95_ (min)	180.5
*R*^2^ adjusted	0.9962
SD (mg g^–1^)	2.565
BIC	36.32
General-order	
*q*_e_ (mg g^–1^)	133.5
*k*_N_ (min^–1^·(g mg^–1^)^*n*−1^)	8.570 × 10^–5^
*n*	2.342
*t*_1/2_ (min)	16.06
*t*_0.95_ (min)	206.2
*R*^2^ adjusted	0.9965
SD (mg g^–1^)	2.470
BIC	36.70

The model’s fitness is determined by analyzing both *R*_Adj_^2^ and standard deviation (SD) values.^34,37^*R*_Adj_^2^ values closer to 1.00 and lower SD values indicate a smaller difference between the model and experimental sorption capacities and, therefore, better model suitability.^34^ In this sense, GO kinetics was the most suitable one, indicated by the lowest SD (2.470) and highest *R*_Adj_^2^ (0.9965), among the three studied models. These results prove that the values of q_t_ predicted by the GO model were the closest prediction to the experimentally obtained q_t_.^34^

However, besides *R*_adj_^2^ and SD values, the Bayesian information criterion (BIC) was also utilized to check the best kinetic model.^34^ The values of the BIC are also displayed in Table 1. When the difference between BIC values of one model and (ΔBIC) another is <2, there is no statistical difference among these models.^34^ When 2 < ΔBIC < 6, the model with the lower BIC value has a prospective of being the best-fitted model.^34^ When 6 < ΔBIC < 10, the model with the lower BIC value has a strong possibility of being the best-fitted model.^34^ When ΔBIC ≥ 10, the model that presents the lower BIC value is certainly the best-fitted model.^34^ ΔBIC between PSO and PFO and PSO and GO models were 30.45 and 0.38, respectively. Based on the BIC, there is no remarkable difference between PSO and GO models.^34^ However, taking into account that PSO is the simplest kinetic model, it would be expected that this model could suitably describe the kinetic adsorption data.

One of the reasons for performing the kinetic adsorption study is to realize the time required for the system to attain an equilibrium. However, considering that the different kinetic models present kinetic constant rates with different units, it is not easy to compare these models. Therefore, *t*_1/2_ and *t*_0.95_ are defined as the time to attain 50 and 95% of the saturation of the adsorbent, respectively.^34^ The values of *t*_1/2_ and *t*_0.95_ are displayed in Table 1. Considering that the PSO model better describes the adsorption kinetics, it could be stated that *t*_1/2_ = 16.03 min and *t*_0.95_ = 180.5 min. For performing the equilibrium studies, a time of 200 min was chosen to obtain the equilibrium adsorption isotherms.^34^

The intraparticle diffusion process further evaluates the kinetic process (see Figure 6b). The adsorption dynamics included three stages. The first step can be attributed to the bulk boundary diffusion of the MO molecules through the solution to the external surface of the adsorbent; this stage is longer when compared to the second stage, which may be due to the higher number of active sites that may also offer high resistance to liquid permeation. In the second stage, MO could be adsorbed and diffused into the interior pores and cavities that are present in the APTES-modified biomass until attaining an equilibrium. Finally, the third linear portion corresponds to the MO molecules’ diffusion to the adsorbent material’s small pores until the equilibrium is established. The k_id_ values for each linear section are presented directly in Figure 6. *k*_id1_, *k*_id2_, and *k*_id3_ were 13.32, 3.647, and 0.04592 mg g^–1^ min^–0.5^, respectively, indicating that the first stage was the fastest, and the rate decreased in the following stages.

#### 3.2.3 Equilibrium Studies

The isotherm of adsorption is a useful tool for understanding how the interaction between the adsorbent and adsorbate occurs. It also provides useful insights into the process’s adsorption mechanisms and predicts the maximum adsorption capacity.

**Figure 7:**
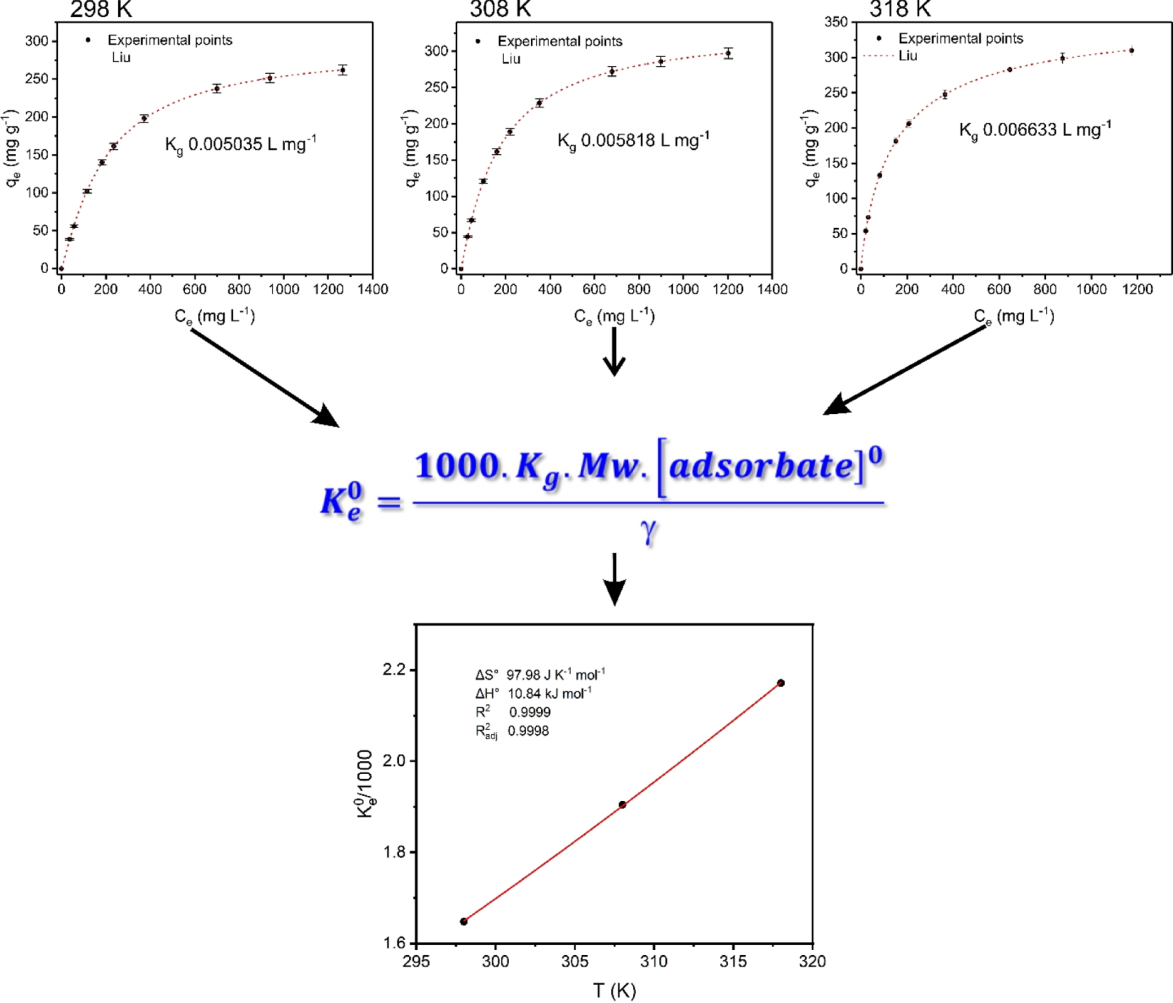
Nonlinear Liu isotherm plots of MO adsorption onto GWW/APTES at different temperatures (K). A contact time of 200 min, an adsorbent dosage of 2.0 g L^–1^, pH 6.0. The obtained Liu equilibrium constant at each temperature was converted to the dimensionless thermodynamic equilibrium constant (). After applying  in the nonlinear van’t Hoff equation, the thermodynamic parameters of adsorption were calculated. For details, see the Supporting Information.

The results of MO adsorption on GWW/APTES were evaluated using Langmuir, Freundlich, and Liu isotherm models.^34^Figure 7 and Table 2 exhibit the equilibrium curves and parameter values, respectively, showing the effective relation between the solute and adsorbent.

**Table 2:** Parameters of the Isotherms of Langmuir, Freundlich, and Sips Models for MO Adsorption on the GWW/APTES Adsorbent

Langmuir	298 K	308 K	318 K
*Q*_max_ (mg g^–1^)	314.6	345.8	340.1
*K*_L_ (L mg^–1^)	0.004296	0.005378	0.007798
*R*^2^_adj_	0.9985	0.9996	0.9986
*SD* (mg g^–1^)	3.620	2.060	4.171
BIC	30.41	19.13	33.24
Freundlich			
*K*_F_ (mg g^–1^ (mg L^–1^)^−1/*nF*^)	15.32	21.03	29.90
*n*_F_	2.442	2.596	2.927
*R*^2^_adj_	0.9541	0.9561	0.9645
*SD* (mg g^–1^)	20.04	22.27	20.71
BIC	64.63	66.75	80.66
Liu			
*Q*_max_ (mg g^–1^)	293.8	334.7	361.8
*K*_g_ (L mg^–1^)	0.005035	0.005818	0.006633
*n*_L_	1.144	1.070	0.8784
*R*_adj_^2^	0.9999	0.9999	0.9999
SD (mg g^–1^)	0.7437	0.1543	0.3496
BIC	–0.2777	–31.73	–15.38

The Liu model was the most suitable model based on *R*_Adj_^2^, SD values, and BIC values because it presented the highest *R*_Adj_^2^, lowest SD values, and lowest BIC values. This means that its theoretical q_e_ values were closer to those found experimentally (Table 2).^34^ The ΔBIC values between Liu and Langmuir and Liu and Freundlich ranged from 30.69 to 50.87 and 64.91 to 94.48, respectively. In this sense, the Liu isotherm model is certainly the best-fitted model.^34^

The Liu isotherm is a combination of the Langmuir and Freundlich models and is suitable for describing both homogeneous and heterogeneous adsorption systems.

By further analyzing the data in Table 2, it can be seen that GWW/APTES displayed very high maximum adsorption capacities of 293.8, 334.7, and 361.8 mg g^–1^ at the temperatures of 298, 308, and 318 K, respectively. These results show that MO removal was affected by the temperature. Further studies on the effect of temperature are presented and discussed later in the thermodynamic studies.^35−37^

#### 3.2.4 Effect of Temperature and Thermodynamic Parameters

To successfully analyze the effect of temperature on MO removal, it is crucial to calculate, evaluate, and discuss the thermodynamic process and its parameters, such as Δ*G*^0^ (Gibb’s free energy change), Δ*H*^0^ (standard enthalpy), and Δ*S*^0^ (standard entropy). Under the studied temperatures, the calculated Δ*G*^0^ and Δ*S*^0^ exhibited negative and positive values, respectively, which suggests that the removal of MO on GWW/APTES was spontaneous and favorable.^35−37^

Thermodynamic parameters for MO removal from GWW/APTES are exhibited in Table 3. The Δ*H*^0^ presented a positive value, suggesting an endothermic adsorption process and that more energy is absorbed from the external environment for the adsorption to take place.^35−37^ The calculated Δ*H*^0^ values were higher than 10 kJ mol^–1^, suggesting that the physical adsorption occurred between MO and GWW/APTES.^35−37^

**Table 3:** Thermodynamic Parameters of MO Removal from GWW/APTES

temperature (K)	*K*_g_ (L mol^–1^)	Δ*G*°(kJ/mol)	Δ*H*°(kJ/mol)	Δ*S*° (kJ/mol K)
GWW/APTES				
298	1.648 × 10^3^	–18.35	10.84	97.98
308	1.904 × 10^3^	–19.34		
318	2.171 × 10^3^	–20.31		

Also, Δ*G*^0^ values are crucial to understanding and explaining the spontaneity of the adsorption process in the thermodynamic evaluation. In this sense, MO removal by GWW/APTES was energetically favorable at a given temperature because Δ*G*^0^ displayed high negative values.^35−37^

By increasing the adsorption temperature, the diffusion rate of MO molecules over the outside boundary layer of GWW/APTES increases, which in turn not only increases the diffusion rate but also speeds up the MO attraction to GWW/APTES surface functional groups.

#### 3.2.5 Adsorption Mechanism

**Figure 8:**
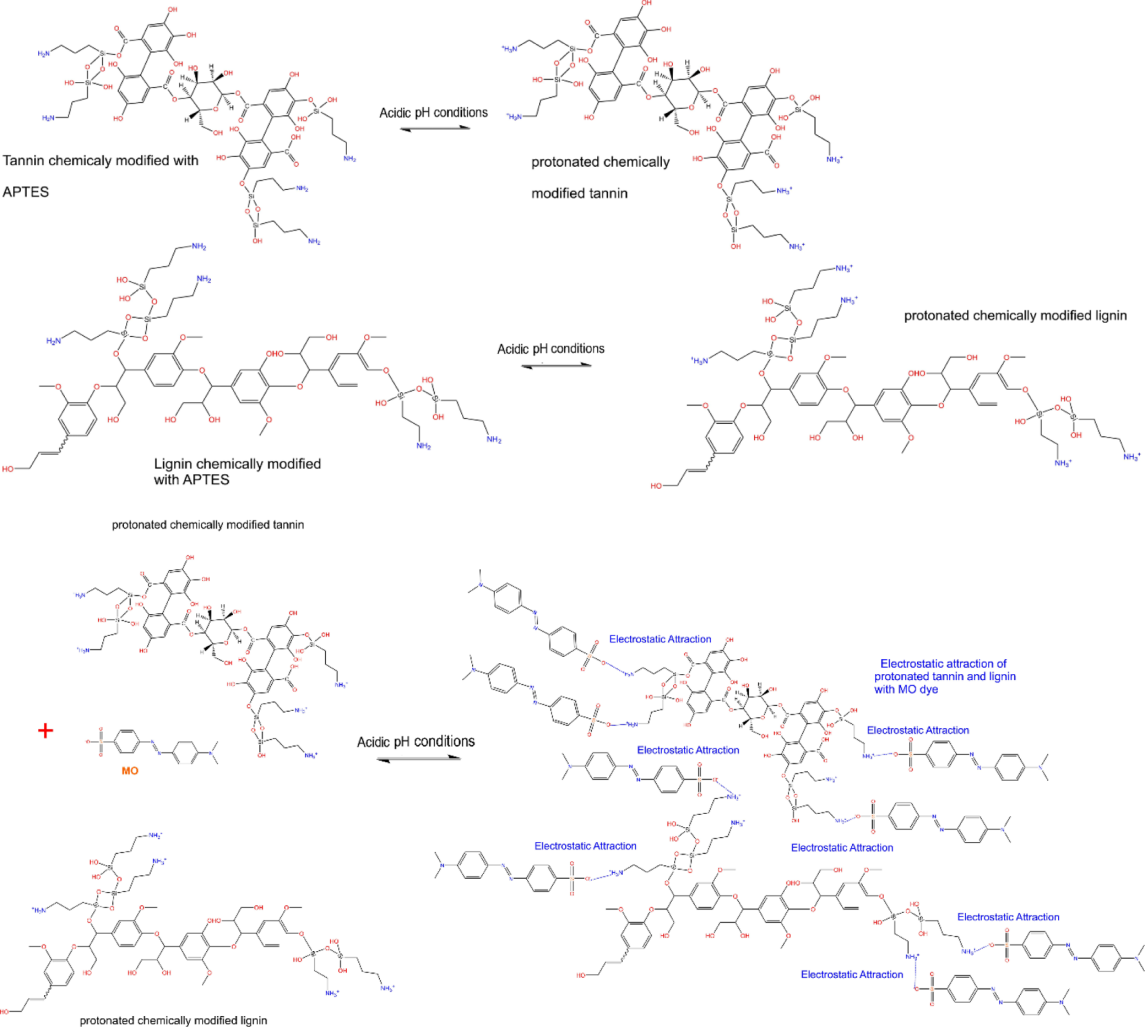
Adsorption mechanism for uptake of the MO dye onto GWW/APTES.

A mechanism of interaction of the MO dye and GWW/APTES could be established based on the physicochemical properties of the adsorbent material (chemical surface and functionalities) and adsorption data (initial pH solution, the kinetics of adsorption, and equilibrium studies) (see Figure 8). The effect of the initial pH of the dye solution highly reinforces the electrostatic mechanism, which was the major contributor to the MO dye removal because its influence was greatly affected by the variation in the pH values.

Looking closely Figure 8, under acidic conditions (pH 2.0), the mechanism of MO removal is based on unprotonated −NH_2_ groups on GWW/APTES that are protonated to form −NH_3_^+^, that is, positively charged groups electrostatically attracting the negatively charged −SO_3_^–^ group on MO dye species.^28,29^ In addition to electrostatic attraction, van der Waals interactions, π–π interactions, and hydrogen bonds can also take place between the other parts of the dye molecules with the biomass portion of the adsorbent contributing to overall adsorption.

#### 3.2.6 Adsorbent Performance: Literature Comparison

The MO dye adsorption tests on GWW/APTES unequivocally presented a satisfactory efficiency of removal. Although the profile of each adsorbent differs from one another, all of them have advantages and disadvantages. Table 4 compares GWW/APTES and other adsorbents reported in the literature studies.^39,44−47,49−55^ The maximum adsorption capacity (*Q*_max_) is the main factor taken into account to evaluate the adsorbent performances. Therefore, the various adsorbents’ performance, including amino-functionalized materials, activated carbons, and clay materials,^39,44−47,49−55^ in the removal of MO are presented in Table 4.^48^

**Table 4:** Comparison of MO Adsorption Capacity and Other Parameters Obtained from the Different Materials Reported in the Literature Studies.^39,44−47,49−55^

material	dosage (g·L^–1^)	Isotherm model	Q_max_ (mg.g^–1^)	refs
iron oxide nanoparticles anchored on amino-functionalized mesoporous silica MCM-41	25	Langmuir	154.23	(39)
N-doped activated mesoporous carbon aerogel from chitosan	0.2	Langmuir	400	(44)
N-doped porous carbon derived from waste cellulose fibers	0.8	Langmuir and Freundlich	337.8	(45)
amine-modified polymers of the intrinsic microporous fibrous membrane	0.4	Langmuir	312.5	(46)
amino group-functionalized UiO-66 MOF	0.4	Langmuir	148.4	(47)
polyaniline powder	1.0	Langmuir	147.0	(49)
Cd-zeolite imidazolate framework	170	Langmuir	145.4	(50)
biochar from grape seeds	20	Freundlich	111.11	(51)
amino-functionalized magnetic bacterial cellulose modified with activated carbon	0.4	Langmuir	103.3	(52)
MnO_2_/biomass from *Terminalia ivorensis*	0.4–40	Langmuir–Freundlich	81.32	(53)
amine-functionalized lignite coal fly ash	0.5–2.5	Langmuir	17.906	(54)
green iron nanoparticles supported on amino-functionalized silica	6.0		9	(55)
GWW/APTES	2.0	Liu	361.8	this work

Among all adsorbent materials highlighted in the table, GWW/APTES displayed the highest *Q*_max_, even higher than that of activated carbon materials,^51,52^ showing high competitiveness in removing color from colorful effluents and possibly many other pollutants from wastewater.

Activated carbon is the most popular adsorbent material because it is characterized by a high surface area, very active free adsorption sites, a porous structure, chemical surface functionalities, and so forth; even so, GWW/APTES presented better efficiency.

Since biomass is a low-cost, readily available adsorbent material, it can therefore be a replacement for more expensive adsorbents in the treatment of water.

#### 3.2.7 Cyclability Test of GWW/APTES

**Figure 9:**
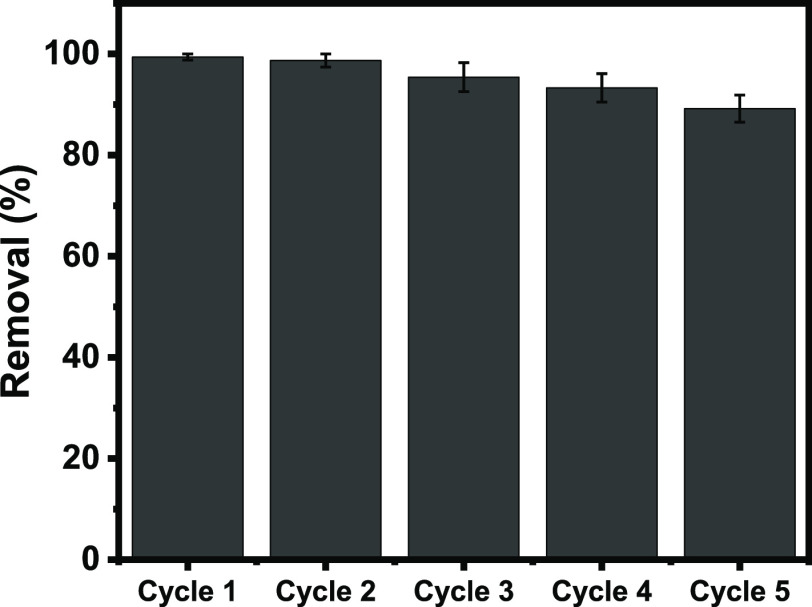
Cycles of adsorption of MO on GWW/APTES.

The cyclability of GWW/APTES using consecutive adsorption–desorption tests was performed according to the methodology described in the study of Lima,^30^ and their results are shown in Figure 9. The MO removal was maximized under acidic conditions (pH of 4.0) based on the pH results. In this sense, 1 M NaOH was used in the desorption of the MO dye onto GWW/APTES. Desorption tests were performed using the same procedure as the adsorption tests. 1.0 g of the MO-loaded material was put in contact with 25 mL of 1.0 mol L^–1^ NaOH solution. The flasks were stirred at 150 rpm for 1 h, and the MO concentration was quantified by UV–vis spectrophotometry. The results demonstrated that 99.9% of MO was released from GWW/APTES. This signifies that NaOH is an effective eluent to desorb the MO dye from the modified material. Four cycles of adsorption–desorption were performed to verify the reusability of GWW/APTES.

The results showed that GWW/APTES presented very effective adsorption–desorption performances. The results of successive adsorption–desorption cycles showed that in the first cycle, a removal of 98.7% was achieved, while after five cycles, a very high percentage of removal was still presented (89.8%). This strongly suggests that the functionalization with APTES provided excellent recyclability to the material, suggesting that it can be used multiple times before being considered useless. This makes the process more sustainable and environment-friendly.

#### 3.2.8 Colorful Simulated Effluent Treatment Tests

**Figure 10:**
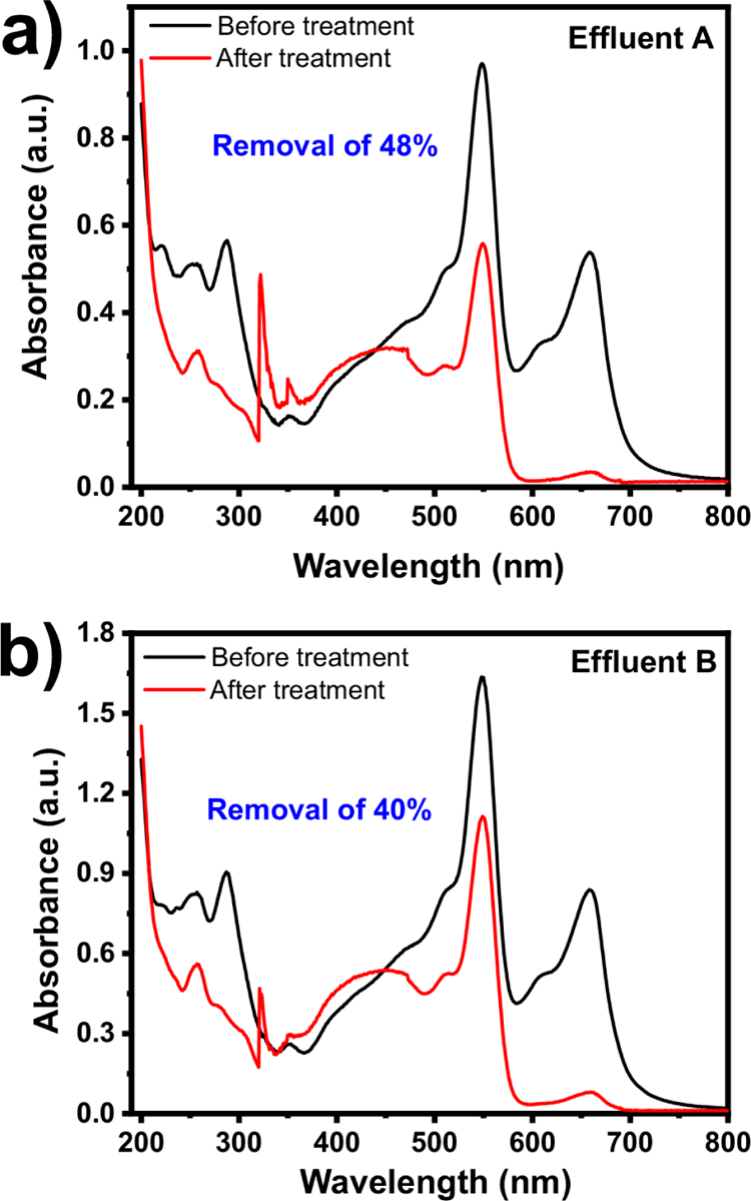
UV–vis spectra of simulated effluents before (in black) and after (in red) the adsorption treatment using GWW/APTES as an adsorbent from Effluent A (a) and Effluent B (b). Conditions: A contact time of 6 h, pH 6.5, 298 K, an adsorbent dosage of 2.0 g L^–1^.

Based on the previous MO dye adsorption experiments, it is expected that GWW/APTES can be applied as an effective adsorbent to treat wastewater polluted with dyes (simulated colorful industrial effluents). Therefore, two simulated wastewater samples with five different dyes (see Supporting Information, Table S1, for details of the used dyes and their composition) were employed to test the ability of GWW/APTES in decoloring them (see Figure 10).

The overall percentage removal was calculated considering the area under the curve of the UV–vis (190–800 nm) spectra of the two synthetic effluents before and after the treatment^23,26,28,56−58^ (see Figure 10). The results exhibited satisfactory percentage removals for both effluents: Effluent A’s and Effluent B’s percentage removal was 40.0 and 48.0%, respectively. Therefore, these results support the practical application of the APTES-functionalized grape biomass material in treating colored wastewater.

## 4 Conclusions

A functionalized biomass GWW was grafted with APTES (GWW/APTES), yielding an innovative and efficient adsorbent material to remove MO dye and colorful industrial simulated effluents. The physicochemical characterization suggested remarkable differences between GWW and GWW/APTES adsorbents, suggesting the success of the APTES-functionalization method. GWW/APTES was successfully employed as an adsorbent material to remove MO dye.

The physicochemical characterization results of various analytical techniques such as SEM-EDS, FTIR, XRD, XPS and point of zero charges have demonstrated that the incorporation of APTES on the GWW structure was accomplished, yielding a functionalized material (GWW/APTES).

The functionalized material showed very effective MO removal due to its unique characteristics, such as an abundance of functional groups on its surface. The adsorption process suggests that the electrostatic interactions were the main acting mechanism of MO dye removal. The regeneration performance revealed that the APTES-functionalized biomass material was easily recycled and reused and showed 89.8% of the removal performance after five cycles.

The modified adsorbent successfully treated two synthetic effluents, which attained a removal percentage of up to 48%. At last, the APTES biomass-based material may find significant applications as an multifunctional adsorbent and can be used further to separate pollutants from wastewater..

## Supporting Information Available

Further description of the kinetic, equilibrium, and thermodynamic models; quality control of the adsorption tests; effluents’ compositions; EDX mapping results of elements for GWW-APTES; and XPS compositions (at %) of GWW and GWW/APTES (PDF)
