# Supplementary material for: 3-Aminopropyl-triethoxysilane-Functionalized Tannin-Rich Grape Biomass for the Adsorption of Methyl Orange Dye: Synthesis, Characterization, and the Adsorption Mechanism
Source: ACS Omega. 2022 May 23;7(22):18997–9009. doi: 10.1021/acsomega.2c02101 (PMC9178721; doi:10.1021/acsomega.2c02101)
Supplement: Supplementary file 1 — ao2c02101_si_001.pdf [file ao2c02101_si_001.pdf]

3-aminopropyl-triethoxysilane-functionalized tannin-rich grape biomass for the Adsorption of methyl Orange dye: Synthesis, Characterization, and Adsorption Mechanism

Edmo H. M. Cavalcante[1], Iuri C. M. Candido[1], Helinando P. de Oliveira[1], Kamilla Barreto Silveira[1], Thiago Víctor de Souza Álvares[1], Eder C. Lima[2], Mikael Thyrel[3], Sylvia H. Larsson[3], Glaydson Simões dos Reis[3*]

[1] Institute of Materials Science, Federal University of Sao Francisco Valley, Juazeiro 48920-310, BA, Brazil

[2] Institute of Chemistry, Federal University of Rio Grande do Sul (UFRGS), Av. Bento Gonçalves 9500, Porto Alegre, RS, Brazil

[3] Swedish University of Agricultural Sciences, Department of Forest Biomaterials and Technology, 90183 Umeå, Sweden

***Correspondence: glaydson.simoes.dos.reis@slu.se**

Supporting Information

Studies of adsorption kinetics, equilibrium and thermodynamic

The pseudo-first-order (Eq 3), pseudo-second-order (Eq 4), and general-order (Eq 5) models [18,20,22,23] were fitted to the kinetic data.

$$q_t = q_e.[1 - \exp(-k_1.t)] \quad (3)$$

$$q_t = \frac{k_2.q_e^2.t}{1 + q_e.k_2.t} \quad (4)$$

$$q_t = \left(q_e - \frac{q_e}{\left[k_N.(q_e)^{n-1}.t.(n-1) + 1\right]^{1/(1-n)}}\right) \quad (5)$$

where t is the contact time (min); q_t, q_e are the sorption capacities at time t and the equilibrium, respectively (mg g^{-1}); k_1 is the pseudo-first-order rate constant (min^{-1}); k_2 is the pseudo-second-order rate constant (g mg^{-1} min^{-1}); k_N is the General-order constant rate [min^{-1}.(g mg^{-1})$^{n-1}$)], n is the dimensionless general-order exponent.

The Langmuir (Eq 6), Freundlich (Eq 7), and Sips (Eq 8) isotherm models; were utilized to fit the equilibrium data [18,20,22,23].

$$q_e = \frac{Q_{max}.K_L.C_e}{1 + K_L.C_e} \quad (6)$$

$$q_e = K_F.C_e^{1/nF} \quad (7)$$

$$q_e = \frac{Q_{max}.K_S.{C_e}^{nS}}{1 + K_S.{C_e}^{nS}} \quad (8)$$

C_e is the MO concentration at equilibrium (mg L^{-1}); q_e is MO sorption capacity at equilibrium (mg g^{-1}); Q_{max} is the maximum sorption capacity of the GWW-APTES material (mg g^{-1}); K_L is the Langmuir equilibrium constant (L mg^{-1}); K_S is the Sips equilibrium constant (L mg^{-1})$^{1/nS}$; K_F is the Freundlich constant [mg.g^{-1}.(mg.L^{-1})$^{-1/nF}$]; n_S (Sips) and n_F (Freundlich) are the dimensionless exponents.

Thermodynamic studies for the MO dye adsorption onto GWW/APTES were performed at temperatures ranging from 298 to 318 K.

The Gibb's free energy change (ΔG°, kJ mol^{-1}), enthalpy change (ΔH°, kJ mol^{-1}), and entropy change (ΔS°, J $mol^{-1}K^{-1}$) were evaluated with the aid of Equations 9-12, respectively [18,20,22,23].

$$\Delta G^{\circ} = \Delta H^{\circ} - T.\Delta S^{\circ} \quad (9)$$

$$\Delta G^{\circ} = -RT.LnK_e^{\circ} \quad (10)$$

$$K_e^{\circ} = \frac{(1000.K_g.\,Mw.\,[adsorbate]^{\circ}}{\gamma} \quad (11)$$

The combination of Equations 9 and 10 leads to equation 12

$$LnK_e^{\circ} = \frac{\Delta S^{\circ}}{R} - \frac{\Delta H^{\circ}}{R}.\frac{1}{T} \quad (12)$$

R is the universal gas constant (8.314 J K^{-1} mol^{-1}); T is the absolute temperature (Kelvin); M_w is the molecular weight of the adsorbate (g mol^{-1}), $[adsorbate]^{\circ}$ is the standard molar concentration of the adsorbate, which by definition is 1 mol L^{-1}; γ is the activity coefficient of the adsorbate. K_e° is the thermodynamic equilibrium constant, calculated according to equation 16. K_e° is dimensionless [18,20,22,23].

K_e° is calculated by converting K_g values (Liu equilibrium constant) or K_L (Langmuir equilibrium constant), expressed in L mg^{-1} into L mol^{-1}. Firstly, the value K_g or K_L is multiplied by 1000 (mg g^{-1}), and then multiplied by the molecular weight of the adsorbate (g mol^{-1}) and by the standard concentration of the adsorbate (1 mol L^{-1}) and divided by the activity coefficient of the adsorbate (γ- dimensionless) [22-24,45,46]. It is assumed that the solution is sufficiently diluted to consider that the γ is unitary [22-24,45,46]. Making these calculations, K_e° becomes dimensionless [22-24,45,46].

The quality control of adsorption data is further described elsewhere [18,20,22,23]. Nonlinear fitting of kinetic and equilibrium data was performed using the Microcal Origin 2020 software. The nonlinear fitting was obtained using the Simplex method and the Levenberg–Marquardt algorithm for performing this task. The adequacy of the kinetic and equilibrium models was statistically assessed employing the adjusted determination coefficient (R^2_{adj}) and the standard deviation of residues (SD) [18,20,22,23] shown in equations 14 and 15 below.

$$R^2_{adj} = 1 - (1 - R^2).\left(\frac{n-1}{n-p-1}\right) \quad (14)$$

$$SD = \sqrt{\left(\frac{1}{n-p}\right).\sum_{i}^{n}(q_{i,\,exp} - q_{i,\,model})^2} \quad (15)$$

where $q_{i,\,model}$ is the individual model sorption capacity expected by the model; $q_{i,\,exp}$ is the individual experimentally measured *sorption capacity*; $\overline{q}_{i,exp}$ is the average of all measured experimental *sorption capacities*; *n* is the number of experiments performed; *p* is the number of model parameters.

The R^2_{adj} and SD values were used to compare different kinetics and equilibrium models. The best-fitted model would present the R^2_{adj} closer to 1.00 and the lowest SD values [18,20,22,23].

Application to synthetized effluents

Two effluents were made, mixing seven dyes to simulate dyes-composed effluents. The different compositions of these synthesized effluents are reported in Supplementary Table S1 (see supplementary data). In order to prove the ability of the GWW-APTES for treating real effluents, a calculation of the percentage removal is done considering the UV–vis spectra area of the two effluents before and after the treatment under the band of the spectra [22-24].

Table S1. Effluents' composition

Compounds	Concentration (mg L^{-1})		λ_{max} (nm)
Effluent	A	B	
Methyl Orange	20	40	522
Reactive orange 16	20	40	494
Methylene Blue	20	40	668
Methyl Red	20	40	507
Rose Bengal	20	40	549
Methylene Blue	20	40	668
Rhodamine B	20	40	544

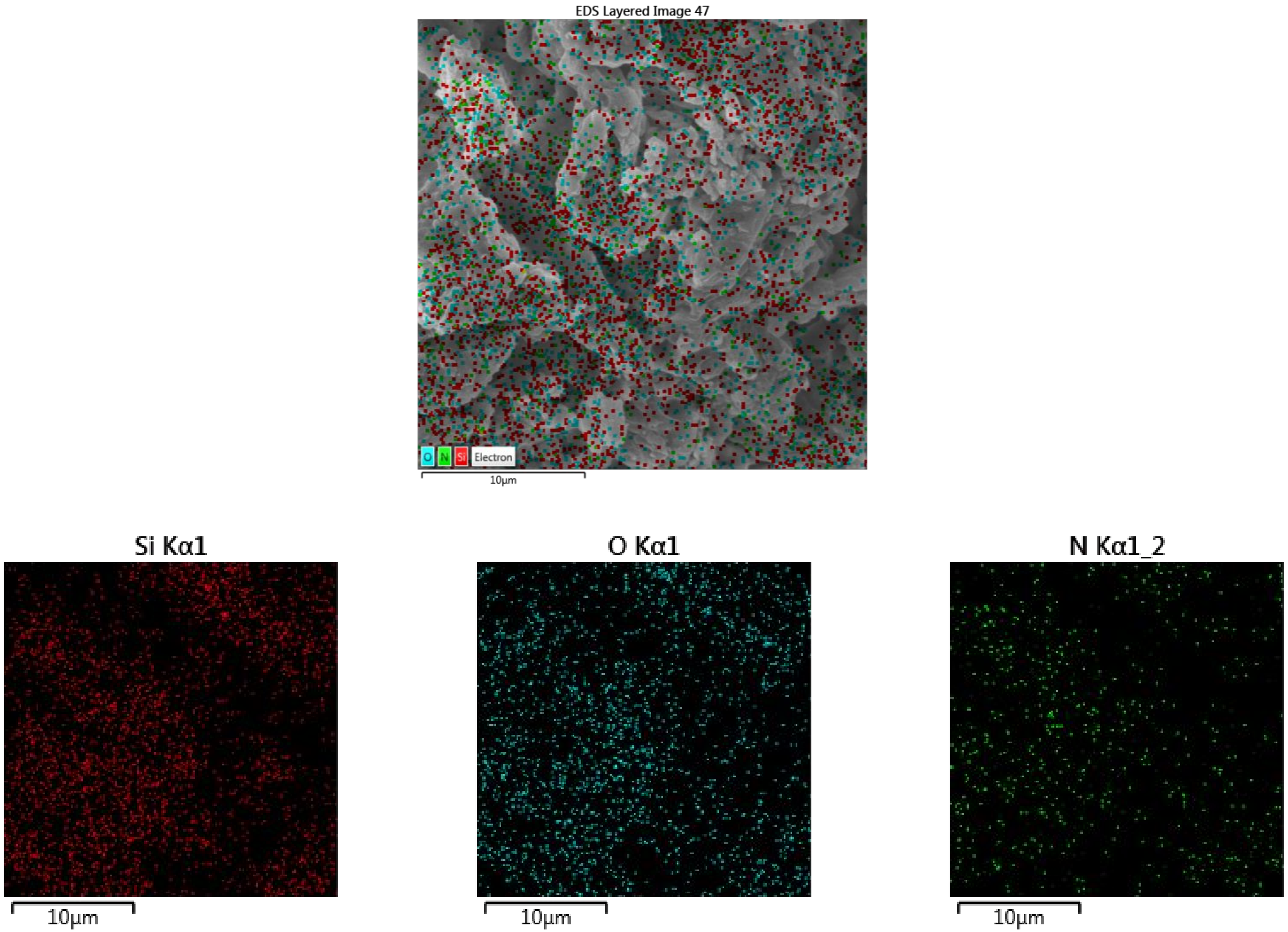

Figure S1. EDX mapping of elements for GWW-APTES (Si = silicon), (0 = oxygen) and (N = nitrogen)

Table S2. XPS composition (at%) of GWW and GWW-APTES

	Elements			
Samples	C1s	O1s	N1s	Si p^2
GWW	76.1	21.2	2.7	-
GWW-APTES	69.5	22.8	3.4	4.3
